# Stage-Based Communication Rehabilitation in Amyotrophic Lateral Sclerosis (ALS): A Review of Strategies for Enhancing Quality of Life

**DOI:** 10.26502/aimr.0230

**Published:** 2025-12-19

**Authors:** Mark C. Jackson, Rafaelle B. Azarraga, Marcel P. Fraix, Devendra K. Agrawal

**Affiliations:** 1 Department of Translational Research, College of Osteopathic Medicine of the Pacific, Western University of Health Sciences, Pomona, California 91766 USA; 2 Department of Physical Medicine and Rehabilitation, College of Osteopathic Medicine of the Pacific, Western University of Health Sciences, Pomona, California 91766 USA

**Keywords:** Alternative and Augmentative Communication (AAC), Amyotrophic Lateral Sclerosis (ALS), Brain-computer interface (BCI), Bulbar dysfunction, Communication, Dysarthria, Motor neuron disease, Quality of Life (QOL), Rehabilitation, Speech-language Pathologist (SLP), Staged based approach

## Abstract

Amyotrophic Lateral Sclerosis (ALS) is an incurable progressive degenerative neuromuscular disease. One way ALS affects patients is through dysarthria significantly impacting a patient’s quality of life by affecting their ability to communicate. This makes maintaining relationships, identity and autonomy difficult, all of which affect psychological wellbeing - a determinant of the quality of life. Dysarthria makes communication difficult, and because the regions affected by ALS first are different for each patient, creating strategies for rehabilitating communication can be challenging. In this review we explore the different communication rehabilitation options available and organize them based on if they are usable based on the onset of intelligibility and locked in state. Interventions before the onset of intelligibility in the early stage are proactive measures such as voice banking and education which empower patient autonomy and a sense of control. Interventions between onset of intelligibility and the locked-in state in the middle stage are alternative and augmentative communication strategies varied in accessibility and usability in patients based on their preferences and functional ability. Late-stage interventions which work after a patient with ALS has entered a locked-in state, are the most technologically advanced alternative and augmentative communication devices and rehabilitate function inaccessible by other methods in this disease stage. While assessing patient values and recommending interventions which meet patient needs is most important in rehabilitation of communication in patient with ALS, using a stage-based approach to evaluate and recommend the treatment of dysarthria and communication rehabilitation will optimize quality of life throughout the progression of disease.

## Introduction

**Figure 1:**
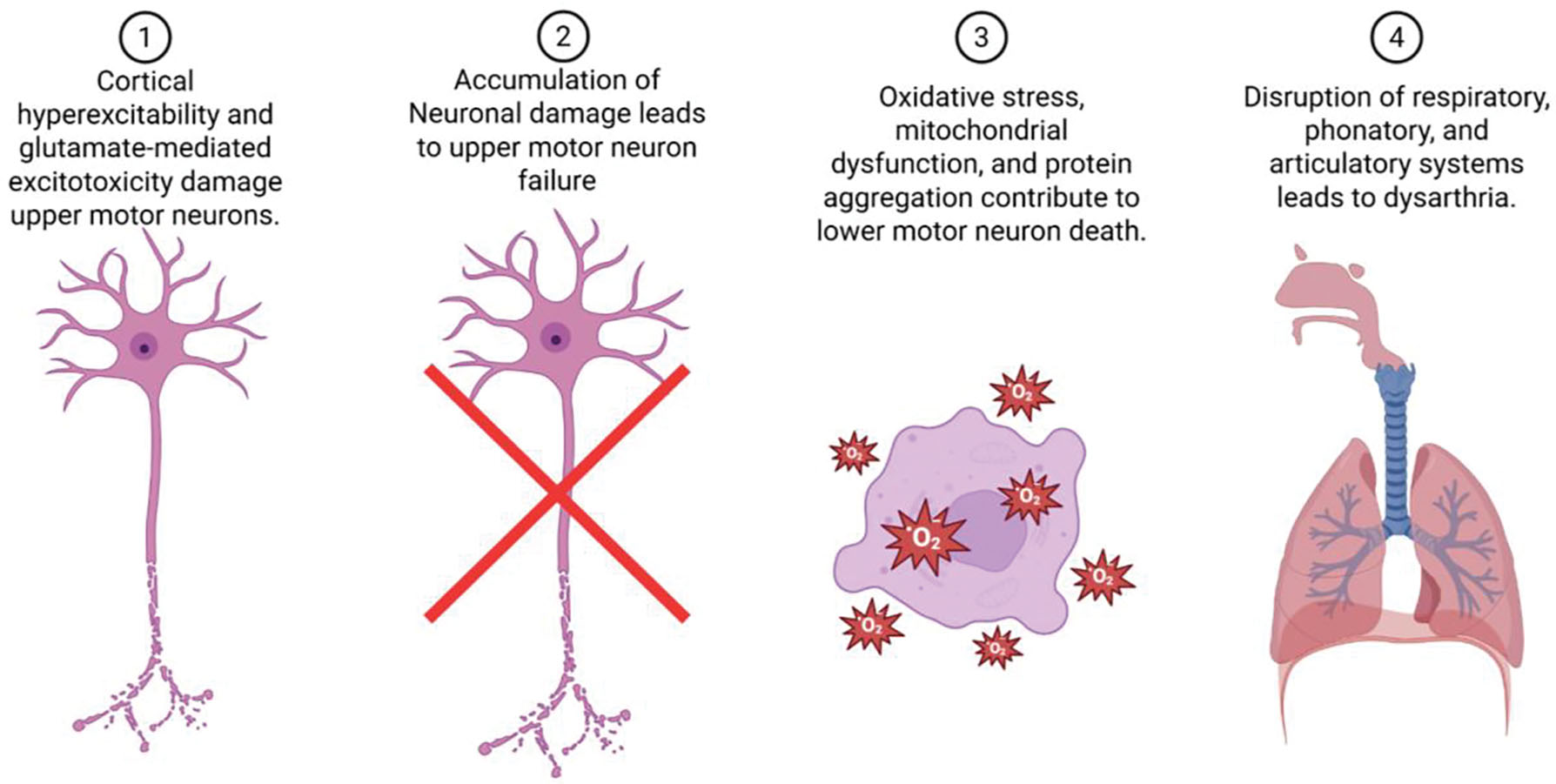
Pathophysiology of ALS is a convergence of several neurotoxic pathways leading to cell death and ultimately motor dysfunction.

Amyotrophic Lateral Sclerosis (ALS) slowly takes away the body’s ability to move, but for many people the loss they feel most immediately is the loss of their voice. The disease targets motor neurons throughout the nervous system, instigating muscle weakness, tightness and ultimately paralysis. When this process reaches the pathways that control speech and swallowing, the corticobulbar tracts, words begin to come out slurred, effortful, or not at all. Dysarthria in ALS arises from the combined failure of cortical and bulbar motor neurons. Several cytotoxic pathways have been identified in the pathophysiology of ALS (Figure 1). Cortical hyperexcitability and glutamate-mediated excitotoxicity damage upper motor neurons, while oxidative stress, mitochondrial dysfunction, and protein aggregation contribute to lower motor neuron death. This dual mechanism disrupts the precise coordination of respiratory, phonatory, and articulatory systems, producing the mixed spastic–flaccid speech pattern characteristic of ALS (Figure 1). In most cases, speech intelligibility falls below 90% within 18 to 24 months of symptom onset, and complete anarthria, the loss of natural speech, typically occurs by the third year, though variation depends on phenotype and respiratory involvement [1–2].

Approximately 25% of patients present with corticobulbar-onset ALS, in which dysarthria often appears as one of the earliest symptoms [3–4]. These patients may notice subtle speech changes, hoarseness, slowed rate, or nasal quality, before any limb weakness emerges. Progression is typically rapid, with life expectancy averaging two to three years from symptom onset [1]. The early involvement of bulbar pathways leads to early challenges in communication, swallowing, and airway protection, demanding immediate interdisciplinary care and augmentative communication support. As bulbar and respiratory neurons degenerate, respiratory failure usually follows within 2 to 5 years of diagnosis, marking the leading cause of mortality in ALS [1]. In contrast, about 75% of patients present with limb-onset ALS, where dysarthria evolves more gradually [3–4]. Speech deterioration in this phenotype may take months or even years, developing as the disease spreads rostrally to involve the corticobulbar system. These individuals generally have a longer survival, with an average life expectancy of three to five years [1]. The delayed onset of dysarthria allows a window for early intervention and adaptation; time to establish alternative communication systems, optimize respiratory support, and maintain autonomy as long as possible. In late stages, patients may enter a locked-in–like state, maintaining full cognitive awareness but losing all voluntary motor and speech capacity [5–6].

Though the pace of progression differs, the emotional and existential impact of dysarthria remains universal. As ALS strips away motor function, speech becomes one of the last frontiers of independence. Preserving communication, through early recognition, adaptive technologies, and compassionate interdisciplinary care, is therefore not only a clinical goal, but a human one.

ALS is a progressive and incurable disease. As such current approaches to treatment are targeted at maintaining function and maximizing quality of life [7]. However, studies have shown that for the most part the progressive physical decline has little or no effect on perceived quality of life in patients with ALS, and instead is more dependent on psychological characteristics, social support and spirituality [8–10]. Speech impairment is the exception to this rule. In one study of several neurological diseases all patients with ALS were found to have a significant psychosocial impact related to dysarthria and intelligibility [11]. In addition, another study corroborated this and found that early speech impairment and complete loss of speech were associated with a lower rating of Quality of Life [10]. Speech is important in the maintenance of relationships which are important to the preservation of identity in patients with ALS [12].

The current standard of care as established by the American Academy of Neurology and European Academy of Neurology utilizes early assessment and multidisciplinary care as well as a variety of augmentative and assistive communication devices to preserve participation in activities of daily living and maximization of quality of life in patients with ALS. However, other than stressing the importance of regular assessment and early referral to a speech language pathologist and a multidisciplinary clinic, there are no recommendations about timing or optimization for treatment of dysarthria and communication rehabilitation [7,13]. Augmentative and Alternative communication devices have been widely accepted and have been reported to maintain patient agency and a feeling of control [12,14]. Due to the progressive and heterogeneous nature of ALS there are dynamic levels of effectiveness, frustration and training associated with different rehabilitation modalities.

Continuous monitoring, and evaluation of communicative function in ALS is paramount to optimizing communication in patients with ALS, and the needs specific to communication are not captured well by measures of overall physical function. The ALSFRS-R is a commonly used questionnaire and has been shown to have a longstanding utility in predicting survival time and helping clinician make decisions about assisted ventilation, nutrition state and respiratory function [15–16]. In one study looking at the current practice patterns in a group of primarily university-based ALS clinics the ALSFRS-R was the only parameter which was routinely collected in 90% of respondent sites [17].

**Figure 2:**
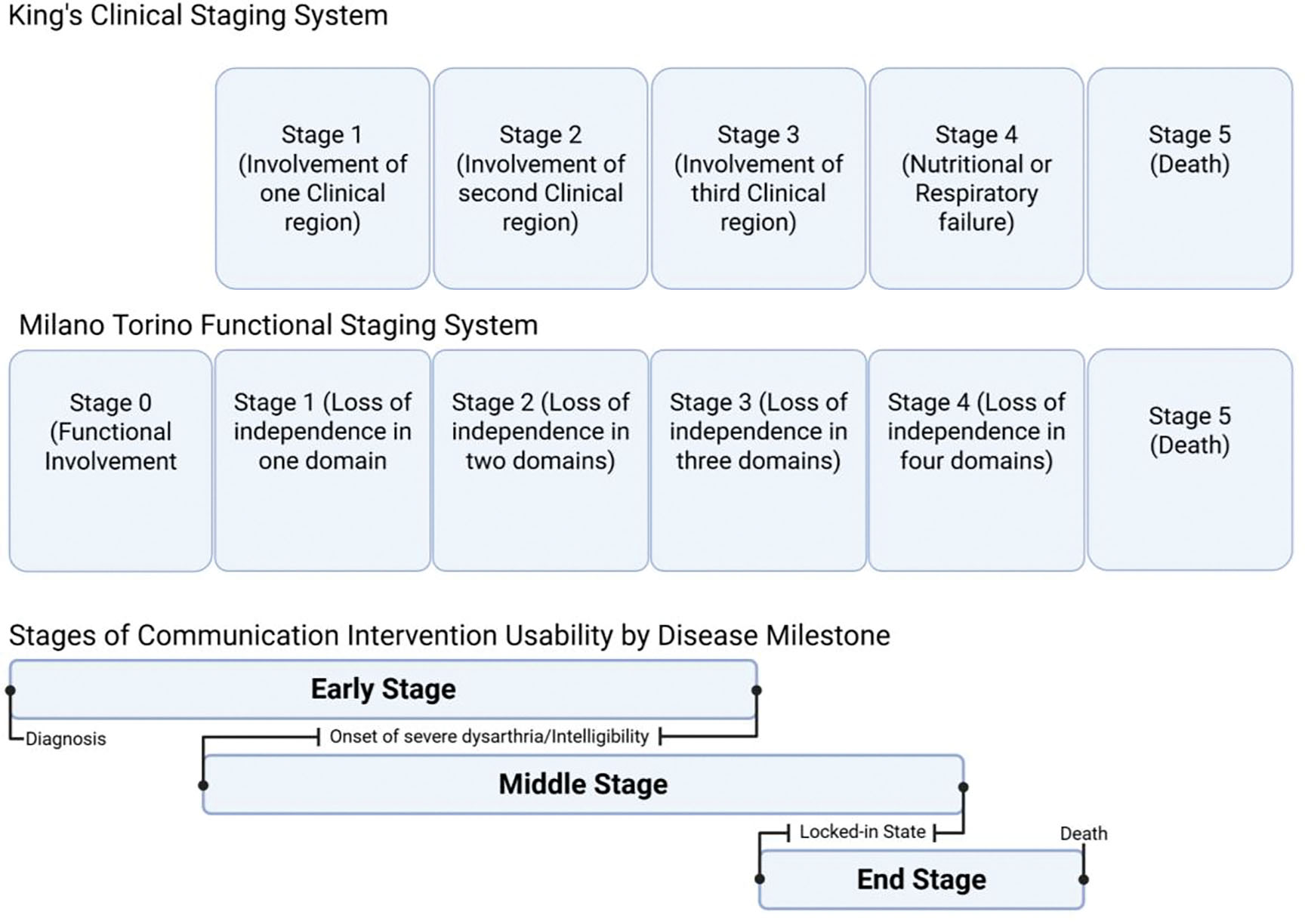
**King clinical staging system** clinical regions include bulbar, upper limbs, lower limbs. **Milano Torino Functional Staging System** domains based on ALSFSRS-R sub scores, independence based on threshold. Domains include walking/self-care, swallowing, communicating, breathing. Stage ranges compared to Kings and Milano Torino staging systems indicate variability in disease milestone timing in different types of ALS such as limb onset vs bulbar onset.

Staging systems such as the Kings staging system and the Milano-Torino staging system help to further delineate key milestones in anatomical and functional ALS progression respectively (Figure 2).

The combined uses of staging systems help to give an objective measure of disease progression and the key milestones affecting communication such as the development of intelligible speech and functional communication disability in motor domains necessary to guide clinicians on timing of different compensatory assistive communication strategies [16,18]. This review seeks to look at the effects of communication rehabilitation strategies in patients with ALS based on their effects on quality of life (QOL) and is organized using the key disease milestones captured by complementary King’s and Milano Torino staging (Figure 2).

## Early Proactive Interventions

The early interventions of ALS are most optimal during the period between diagnosis and initial presentation of bulbar symptoms. The initial presentation of ALS is most commonly in the upper limbs, with little to no deficit in bulbar function. In this period communication can be done in most patients with ALSs through the largely preferred method of one’s own speech without reliance on augmentative and assistive communication devices [19]. This is also a great time to educate patients with ALS and proactively prepare for disease through modalities such as voice banking which are optimal before onset of intelligibility [20]. Strategies for maintaining quality of life in this stage should include preserving natural voice thereby maximizing communicative participation, a social determinant of quality of life [21]. One such strategy is early referral to a speech-language pathologist (SLP) who can prepare patients with speech therapy and assess communication device needs. Early introduction to Augmentative and Alternative Communication (AAC) has shown to be beneficial for psychosocial outcomes [22–24]. Introduction at diagnosis to augmentative and alternative communications leads to better existential and psychological outcomes as compared to delayed intervention, in the short term and decreased decline of outcomes in the long-term [23].

As discussed before, autonomy is a factor influencing quality of life in patients with ALS. Education of patients to disease processes and treatment allows patients to maintain this autonomy through informed decision-making giving patients a sense of control [12]. Lack of education has been identified as a prominent barrier and enabler in the treatment and management of dysarthria in pwALS [25]. In a survey on the use of AAC in patients with ALS (pwALS), less than 50% reported that their family members or other important people received education or support related to communication for pwALS [26]. Education of communication partners is vitally important as many pwALS only wanted to communicate with a few close people, rated maintaining social closeness and relationships with friends and family was high priority, and were most interested in communication training with frequent communicators. [12, 27–28] In interviews with pwALS, most participants reported that they perceived providers as avoiding conversations about the late stages of ALS and noted that they thought that the knowledge is a good defense against challenges later in the disease allowing for proactive measures such as voice banking [29].

Proactive use of voice banking can help maintain a sense of identity later in the disease process. Voice banks can be played back to directly play recordings of the voice of a pwALS, as well as be used in production of voice synthesis. In a survey of patients who decided to voice bank the data suggested that preserving identity was the main motivation to use voice banking and that it would help to preserve social networks [30]. While many patients had heard of voice banking, not many patients used it [26]. Early implementation of voice banking is paramount if a patient is interested as it requires some preparations requiring the recording of 300–500 sentences or phrases to create personalized models [29, 31–32]. These models could either be used to train artificial intelligence to synthesize a voice or to be played directly. pwALS who were surveyed about preferences for generated voice make up agreed that they would want a synthetic voice to sound as close to their natural voice as possible, and showed this was a high priority alongside being able to be understood as they preferred voices similar to natural voice which is only frequently understood, to a voice that is different from natural voice and almost always understood [20]. New methods for voice banking are adopting principles which maximize ownership, preservation of identity, dynamic, collaborative and personal processes which give hope. These methods use traditional voice banking in addition to synthesis of voice [33]. Reasons patients may not want to use voice banking include its constant reminder of the progressive nature of ALS, significant speech impairment, inaccuracy of synthesized models and inability to replace their natural voice [30, 34].

Prompt referral to speech language pathologist and multidisciplinary care centers is a consistent recommendation in the management of dysarthria in ALS [7]. Speech language pathologists are often the provider responsible for the evaluation of a patient’s speech and are paramount in determining what communicative strategies would be effective in ALS. Speech therapy has shown effectiveness in a study comparing ALSFRS-R score deterioration rate before and during the Covid-19 pandemic - a period of no access to speech rehabilitation services. The period of no therapy showed a more significant deterioration in ALSFSRS-R score [35]. One of the interventions SLPs provide is in helping a patient to maintain their own speech to communicate which has been shown to be a preference and a primary mode of communication for many pwALS [28, 36]. Compensatory strategies such as slowing down sentences for listener comprehension and choosing quiet spaces to speak are most effective in this regard. In contrast, speech therapy focusing on increasing strength of vocal muscles is not recommended in the management of dysarthria in ALS [37]. Despite the benefit SLPs provide to patients, one barrier is that choosing when to go to SLP was important for patients’ feeling of control, especially while dependency on rehab services was perceived negatively [38]. In addition, while speech therapy had a positive influence on reported QOL in pwALS, communication devices were found to have a higher impact on reported QOL [22]. SLPs and communication devices are not separate and SLPs play a large part by recommending different communication devices to pwALS. Despite this role, some pwALS reported difficulty with training and SLP unfamiliarity with AAC use, and that for several participants SLP intervention was focused on swallowing rather than their communication [36, 38]. Overall, the decision of a patient to see a speech language pathologist is a key and impactful step in management of dysarthria in pwALS and is an important step in utilizing AACs.

Early introduction to augmentative and alternative communication has been shown to have consistent improvements in QOL compared to later introduction [29]. In one Study of pwALS bulbar onset who were separated into two groups one where patients were referred an AAC device at baseline, and the second referral for AAC when ALSFRS-R bulbar sub- score 1 or 0 (meaning speech requiring nonvocal communication or total loss of useful speech). Early intervention showed increases in QOL measures (existential and psych measures of QOL) from baseline at 3–4 months, while the latter group declined in QOL measures. While both groups declined in the McGill QOL psych and existential measures at 7–10 months, the early intervention groups measured psychological QOL was significantly higher than later intervention [23]. Furthermore, early introduction could serve as a training period for pwALS to seamlessly transition to AAC as a primary communication method [29]. In a study of patients using a three phase AAC intervention model, an AAC training period was conducted by a speech therapist and neuropsychologist for the first three months where the patient’s language ability was monitored, and needs and desires for future communication were assessed. After the training period, a familiarization phase began where a tailored combination of low-tech AAC such as paper tables with alphanumeric symbols or preformed sentences, and pictorial tables to describe pain were provided to the participants. At the end of both the training phase and familiarization phase patients reported improvements in quality of life and mood [24]. In the early stage of ALS proactive AAC use can ease transition into later stages of disease.

## Middle Staged Compensatory Interventions

As the ALS disease process progresses, there are varying domains of dysfunction, one key anatomical domain is bulbar dysfunction which contributes to speech and swallowing. The functional counterpart to bulbar dysfunction regarding dysarthria is dysfunction in communication. After the progression of disease past these milestones, speech can become intelligible and fatiguing for patients, and alternative forms of communication are needed to properly communicate. This is where nonverbal communication strategies, and low-tech augmentative and alternative communication devices can be used with less training and difficulty relative to higher tech. As the progression of disease, communication goals and preferred modes of communication can be drastically different for each patient.

In a survey of AAC use in the US 78.7% of respondents used telephone communication and 84.7% participated in video calls and all eligible participants engaged in face-face conversation (26). Many patients only wanted to communicate with few people and rarely strangers [12, 27]. In addition, patients reported Frequent communication partners were better able to understand pwALS speech [28]. In another survey of caregivers for pwALS the highest level of reported use of AAC was for expressing basic needs, but 80% of respondents also said providing the ability to stay connected was a mandatory or desirable function of AAC devices [39]. Acceptance of diagnosis of MND and assistive technology was second highest enabler and number one barrier, one user noted acceptance of assistive technology as reminder to ongoing loss of function, independence [25]. Overall pwALS report many utilized communication modalities and most consistently face-face communication throughout progression of disease, most commonly using communication to stay close to a few close people and to communicate basic needs. Initial recommendations should meet these needs, but should be highly personalized

Following use of natural speech, gestures and writing are found to be the second most utilized communication method in face-to-face interactions until combined use of speech and non-speech communication methods is necessary- ALSFRS-R sub score 1, where it exceeds usage of natural speech [26]. These methods alongside low tech AAC alternatives such as alphanumeric boards and pictorial boards can be classified under partner assisted methods due to the need for a caregiver or listener to visually interpret and confirm meaning from pwALS. pwALS can use alphanumeric and pictorial boards by pointing with their hand or gaze to symbols or numbers in succession to convey meaning. Another method is communication through physical signals such as blinking in morse code, or yes/no communication codes by blinking [13, 40]. Consistently the use of AAC showed great utility in providing agency to pwALS, and low-tech options provide communicative ability for older and technologically illiterate populations which was reported as a barrier to technologically demanding AAC [12]. The easier acquisition of these methods is doubly important in older populations as decline in ALSFRS-R bulbar score is progressively worse with older age of onset [41]. These methods also benefit in being more affordable, readily available and not requiring ongoing support of referral to specialized AAC hub as complex AAC does [7].

The benefits of partner independent communication devices such as speech generating devices are decreased caregiver burden, and greater independence for patient communication. Everyday devices such as smartphones and laptops can be used for generating speech while pwALS retain adequate function to use them. Other purpose-built devices such as light writer and switch-based systems can extend usability in disease progression by optimizing ergonomics for pwALS [42]. In patients with inability to use their natural speech purpose-built Speech generating devices reach above 50% usage in pwALS. In one study pwALS largely reported acceptance of AAC device usage 90% immediately 6% delayed and no one discontinued use of AAC use, with only rejection of devices being due to cognitive impairment from frontotemporal dementia [14]. More technologically compatible devices can allow access to the internet and to modes of communication such as email and social media which has also shown benefit for pwALS increasing communication partners, network of support and opportunities to communicate with important people [43].

Amongst these speech generating devices, microswitches provide an extended usability in progression of disease due to reliance on only one or a small group of muscles. As progression of ALS is different for every patient, a variety of microswitches can be used and personalized for each patient. Different types of microswitches include ones relying on head movements, sound detection, chin movements, and mouth pressure can be used [44–46]. These Microswitches attached to a computer and an interfacing could be used to allow patients with ALS to control a television system, text messaging system, and access to preferred music and videos, which was rated highly in fostering social function by health care providers [47].The need for microswitches can evolve as ALS progresses, sometimes rendering previous microswitch systems unusable. However, after a training session, all patients who had previously lost the ability to use a different microswitch system were successfully able to use the LeMMS system. Furthermore, all patients maintained the ability to use LeMMS at the 6-month follow-up, and 45% were still using it at 12 months (7 patients had died and 3 had switched to Eye-Tracking Communication Devices [ETCD]). The LeMMS system is easily applicable, reliable, and cost-effective, offering a simpler-to-use alternative to other devices and thus postponing the need for more sophisticated technology [42]. Overall, there are a plethora of low tech and higher tech AAC methods with a large range of activation methods, applications and accessibility. These methods depend on specific patient needs and are most useful in different specific anatomical disabilities for viable and optimal use.

## Late-stage interventions

Finally Late-stage communicative options present a vital role in the preservation of communication in ALS especially after a functionally locked in state or administration of intubation for respiratory support. Eye tracking computer systems are included in this category, because ocular movements are usually preserved functions in many patients until the very last stages of disease. Brain computer interfaces are unique in that they may be used without any motor function and are being more researched as there are advancements in technology. They can enable functions which are inaccessible by any other compensatory method. These methods can restore functions that would otherwise be completely unavailable. However, late-stage systems are difficult and tiring to use, often needing extended training periods, and routine technical support. Early adoption and training of these communication modalities in earlier stages of disease should be used to bridge this gap, and routine technical support can be a barrier if a patient is far away from a multidisciplinary ALS clinic or a provider who has the knowledge of using the device. Remote monitoring and telehealth can help to manage this gap as well.

Eye tracking computer systems are very similar to alphanumeric boards and eye transfer boards in that patients can use them by gazing at a certain area of the board or display to communicate meaning. While manual boards require a caregiver to communicate, ETCS are caregiver independent and work by using infrared cameras to track the movement of the cornea and pupil to determine where a patient is looking on the screen. Despite its complex technology ETSC has been shown to be easy to use and effective for most users. When one ERICA eye gaze system was compared to alphabet boards, although patients were faster to communicate with alphabet boards, they rated the ERICA system as easier to use [48]. Similarly, both eye transfer boards and ETCD were shown to increase communicative ability, ETCD was associated with a comparatively greater increase in communicative ability and was shown to have higher user satisfaction [49]. New advancements are continuously increasing the utility of these devices. Advancements such as the implementation of artificial intelligence have been shown to be able to save motor actions compared to traditional predictive keyboards and showed increased text entry rates in two eye gaze AAC users [50]. As technology has developed, use of ETCS has become prevalent in late-stage ALS, allowing for continuous communication through eye movement which is preserved late into disease. In severe disease stages the use of ETSC increases and is rated as indispensable for everyday life in these populations and that communicative abilities were worse when the ETSC was removed [51]. Altogether ETSC enablement of communication in an easy to use and personalized format increases determinants of quality of life. Like broader trends in pwALS [8–10] QOL was largely independent from physical function in a survey of patients in and their next of kin in the locked in state using ETSC [52]. ETSC use consistently has been shown to increase QOL in pwALS [51, 53]. ETSC can help patients to maintain mental autonomy thereby improving psychological wellbeing and quality of life. One group of ETCS users who requested the ETSC device used it between 100–720 hours per day. ETCS in users with consistent low daily less than 120 minutes ETSC utilization per day reported barriers were eye gaze tiredness (72.7%) and oculomotor dysfunction (18.2%) [54]. Ultimately ETSCs are dependent on patient oculomotor abilities. Dependence on motor function is a common denominator for all previously discussed forms of communication including natural speech which can cause excess fatigue in pwALS in whom fatigue is a common symptom.

ETSCs are more widely used and accessible compared to the other late stage AAC- Brain computer interfaces. However, the brain-computer interfaces (BCI) have shown increased accuracy compared to eye tracking and have been reported to be easier to use [55–56]. Most importantly brain computer interfaces are usable without any motor function and can be used throughout the disease. In one patient who used the device for seven years. As the patient’s motor function declined the pwALS progressively lost the ability to use their eye tracking technology. The patients initially used BCI as ancillary communication and then transitioned to use it as their sole communication method [57]. BCIs work by translating neural signals of attempted speech into text and have been shown to be able to instantaneously synthesize voice with preservation of paralinguistic features [58]. Paralinguistic features are the vocal but nonverbal parts of communication such as tone, speed and volume. These features relay key parts of communication and are key parts of personal expression and are a step towards replicating a patient’s natural voice- a highly desirable feature in AAC (20). BCI can enable expression through art when combined with the P300 Brain painting application [59]. In one case study of a woman with long term ALS in the Locked in state used the BCI brain painting application independently with reported improvement of quality of life and high satisfaction. Furthermore, this patient went on to display and sell paintings made with the software [60]. BCIs are standalone in their ability to restore communicative function in pwALS without motor function and is the only AAC device enabling instantaneous speech generation and brain painting as a creative outlet. Despite novel rehabilitation of function, users prioritized accuracy and speed of operation of AAC devices. In a study in 2011 many pwALS expressed strong interest in BCI’s with accuracy greater than 90% satisfying 84% of respondents, and 72% of respondents wanted a speed of operation of 15–19 letters per minute [61]. At the time BCI devices had not provided performance to meet these metrics. Now in 2024 a case study showed that a 45-year-old man with ALS achieved high proficiency using a BCI. Within one day, he demonstrated 99.6% accuracy with a 50-word vocabulary and 90.2% accuracy with a 125,000-word vocabulary. Over 248 combined hours spanning 8.4 months, he maintained an accuracy of 97.5% and a speed of 32 words per minute. Overall, BCIs have been observed to be utilized significantly more than other communication methods when accessible to patients [5]. Barriers to implementation included the invasiveness of BCI electrodes, which can either be placed on the surface of skin or surgically implanted within the brain. In a survey of pwALS at varying points of disease progression most were interested in less invasive procedures, with 84% of pwALS were okay with electrode cap, 72% willing to undergo outpatient surgery and 41% willing to undergo surgery with anesthesia and a short hospital stay [61]. Interestingly, when responses for patients with tracheostomy- a disease progression milestone indicating later stages of disease and those without were compared, patients with tracheostomy showed a mild tendency to be interested in non-invasive BCI [62]. This indicates that BCI development should continue to develop non-invasive options. However, the benefits of invasive intracortical options have been shown to allow users to communicate effectively on the first day with high accuracy [5, 58]. While in contrast non-invasive measures such as the P300 system used for brain painting have shown to be usable for independent home use after six different one-to-two-hour visits [63], increasing from an earlier case that required 7 months to establish independent use in a brain click BCI system [57]. In current BCI users previous barriers to long term independent home were inability to be used by non-technical personnel, lack of convenience for basic communication, lack of customization and difficult implementation of long-distance technical oversight. These barriers were previously overcome through training sessions, optimizations to user interface, and monitoring and repair through internet and mail respectively. In this case BCI was used daily and extensively over 2.5 years and had positive reports for home use. This required four to five separate one hour training sessions for caretakers [63]. Further development in multidisciplinary home care and support has been shown to have high patient satisfaction and caregiver perception of increased quality of life at end of life [64].

## Discussion

ALS is a progressive and incurable disease with few treatment options which only delay progression. Due to its incurable nature, treatment of ALS focuses on improving patient QOL and helping patients to lead fulfilling lives. QOL is independent of physical function with the notable exception of dysarthria and is mainly dependent on psychological factors. Dysarthria leading to impaired communication is a large effector on psychological well-being stripping patient autonomy, identity and community away. Therefore, it is most important to prioritize a patient’s ability to voice their decisions and maintain relationships by preserving communication throughout the disease. Due to the progressive nature of ALS, the bulbar and motor function of the patient varies widely and often affects patients in different ways at different times. In addition, patient preferences and comfortability with certain communication methods change which forms of compensatory or alternative communication are viable. Because of the unique challenges in each pwALS, frequent assessment and evaluation of communication methods should be done. Today, the most common assessment of physical function in patients is the ALSFSRS-R which is most useful in predicting mortality and overall disease progression. However, as a score it fails to capture distinct communication challenges throughout the disease process. Using a combination of staging systems which has been shown to be additive in benefit, we can better capture disease milestones and identify treatment options which would best address patients’ functional needs. A stage-based approach to treating dysarthria and communication dysfunction will help to maximize quality of life in pwALS. While no system should replace individual assessment and clinical judgement in treatment of communication dysfunction in pwALS, we aim to provide a review of available compensatory, alternative and augmentative communication methods and their effects on quality of life to introduce these methods in a timely manner optimizing communication throughout the disease.

In this review using the Milano-Torino and King’s ALS staging system, early interventions were defined as those which are optimally used before the onset of intelligible speech. As ALS is predictable in its linear progressiveness in this stage, assessment, education and proactive training are paramount in preparing patients for later stages of disease. In some patients this gives them a semblance of control improving the patient’s psychological wellbeing. Most importantly, education of the disease process and prognosis is important in including patients in the decision-making process- preserving autonomy and maximizing the assessment of patient values before making recommendations. Education of caretakers and those close to the patient is important as well as most patients are determined to maintain relationships with a small group and education of communication partners especially in AAC or partner assisted communication methods can optimize ease of communication. Further proactive steps like voice banking can help to preserve natural voice and identity and be later incorporated into voice synthesis algorithms or be used as a straight recording. These methods help to preserve identity which is a determinant of psychological wellbeing and quality of life. As natural speech is most patients preferred communication method maintenance and compensatory strategies should be employed to maintain it. Referral to speech language pathologists achieves this as they can teach compensatory behaviors, as well as enabling the assessment of patient values for further communication methods. However, the evidence of the ability of speech therapy to maintain natural speech is weak and limited, and expectations should be related to pwALS as such. Early referral to SLP is still important as it has shown to increase patient QOL and is important in providing personalized recommendations for communication rehabilitation. One form of communication rehabilitation that SLPs can recommend are AAC. Early introduction to AAC has shown a consistent improvement in QOL, as well as overall AAC devices improving quality of life. AAC devices should be referred to liberally as they can be big effectors on communication.

**Figure 3:**
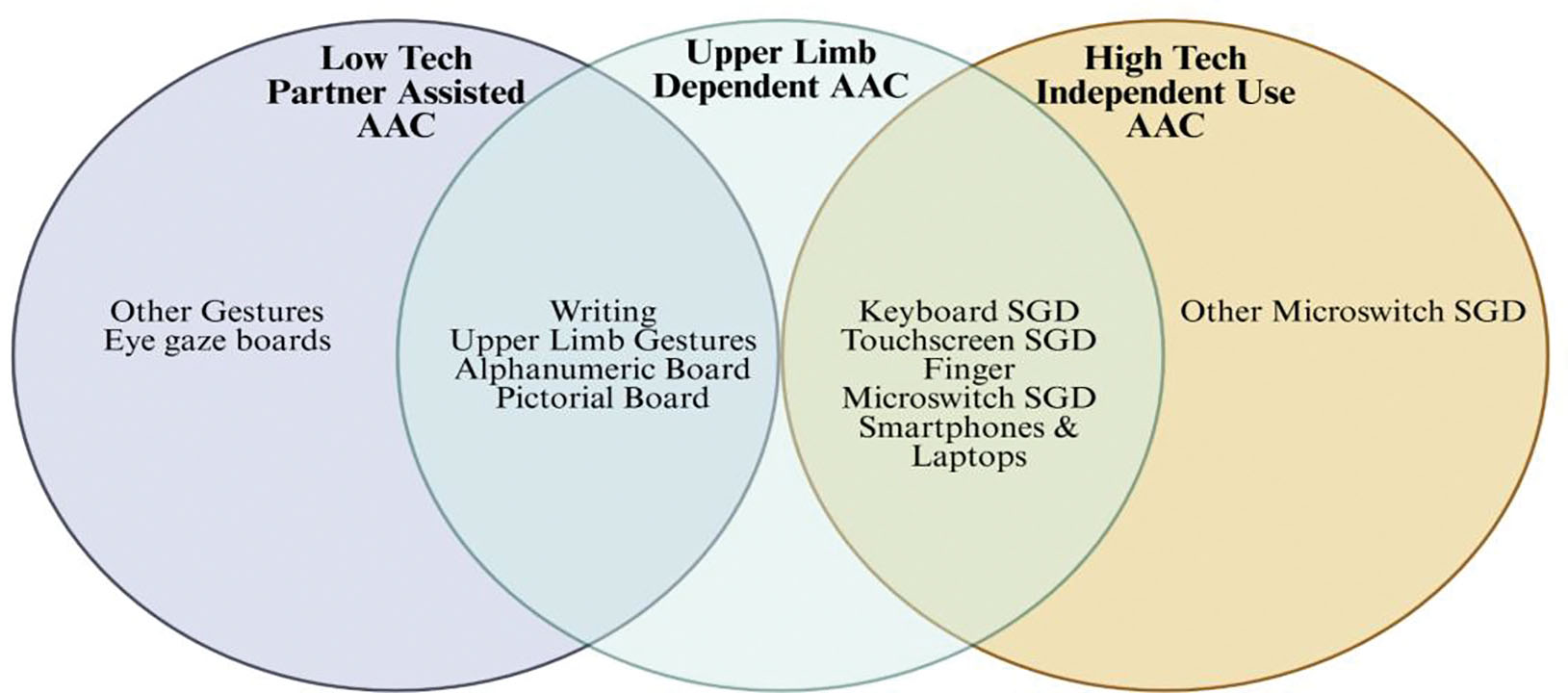
A schematic diagram showing the overlap and different forms of AAC for pwALS. Other gestures include blinking, nodding, etc. The examples of other microswitch speech-generating devices (SGDs) include mouth pressure microswitch, tongue operated microswitch, etc.

Many of the AAC devices fall into the middle stage interventions after the onset of severe dysarthria or intelligible speech as they are independent from the patient’s natural speech (Figure 3). Choosing which AAC to use can vary depending on the patient’s technological literacy, motor domains of dysfunction and personal preference. For example, a technologically illiterate pwALS who cannot control movement of his hand may not be best served by a finger microswitch communication system but may benefit more from a physical eye gaze board. With many different progressions in disease in pwALS and with different preferences and comfortability, the main management decision in the rehabilitation of communication should be which of the many different treatment options to choose and personalize to the patients’ needs. Amongst these low-tech communication methods have the downside of requiring a communicator to focus on interpreting the patient’s communication such as in gestures or in written messages. In contrast high-tech AAC options use programs to generate speech allowing for patients to communicate audibly independently. This both allows a pwALS to communicate with more communication partners if they wish and to exercise more autonomy and independence further contributing to psychological wellbeing and thus quality of life. In addition, caretaker independent speech also reduces burden on caregivers and can ease the strain or bitterness in relationships stemming from over-dependence of a pwALS on their caretaker. Further research should be done into the relationship of ALS management on caretaker quality of life to better address the needs of communities and the healthcare system. High-tech AACs main down sides are that it is more difficult to use or increase training durations in patients with technological illiteracy. In comparison to low-tech AAC like gestures, which are more broadly familiar than technological interfaces, high-tech AAC options can increase friction in communication for pwALS. However, the benefits of greater independence and on caretaker relationships is unique and a positive effect on quality of life and suggest that high-tech AAC options should be offered first when trying to maximize pwALS QOL. Amongst middle stage AAC options there are many intuitive and generalizable types of devices with different usability in different forms of disease such as smartphones and purpose-built SGDs like the light writer system. As many are similar, with minor differences in accessibility for patients with upper limb dysfunction and integration into communication programs we would not expect significant differences on their effects in quality of life in pwALS. Another option includes microswitches which are highly customizable and have been shown to be effective forms of communication throughout the disease process and can be used into the very latest stages of the middle stage. They are also simple to use due to their analog nature and offer a bridge to higher tech AAC methods. Middle stage AACs biggest utility in their ease of use and customization, they can introduce pwALS to AAC methods, and provide simple, fast and cost-effective solutions to AAC. However, they lack the extended viability and range of uses of higher tech AAC methods (Figure 3).

Late-stage communication interventions are the only AAC methods which work into the locked- in state. ETCS have become a mainstay in the communication rehabilitation for pwALS and have shown greater effects on QOL determinants compared to middle stage methods. With ETCS becoming more common and well known, combined with their extended viability into late stages of disease and greater ease of use increases in quality of life, they should be introduced earlier after the onset of natural speech intelligibility in pwALS with middle stage AAC methods offered as a bridge and as an alternative to ETCS. In addition, middle stage AAC methods could be used in combination with ETSC to mitigate the barriers to extended ETCS use in eye gaze fatigue.

Finally, BCIs are the newest and most technologically advanced form of AAC available for communication rehabilitation in pwALS today. While early models saw that they required long training periods and were not accurate or fast enough to meet user expectations, new advancements especially with the introduction of artificial intelligence have made them extremely effective AAC devices. They are unique in that they are usable throughout the disease phase and can restore function which is currently achievable by any other AAC methods such as brain painting or replicating paralinguistic features through speech. These functions further can contribute to a patient’s ability and show great user satisfaction. Unexpectedly some BCI models have been found to have very short training periods, with remarkable accuracy and speed. In addition, advancements in the technological support and the range of multispecialty high tech AAC centers have drastically increased the regions where pwALS could have access and support to these devices. BCIs are not yet a common treatment for ALS and have been showing greater improvements and results in clinical trials, and even more are available commercially for home use. However, when they do become available, they will change the landscape in AAC in ALS as well as in other neuromuscular disorders. Future research should be done to further study the use of BCI in pwALS and to create an easy to implement and accessible AAC method.

Notably this review was limited due to there being inconsistent measures of quality of life and subjectivity in interpreting quality of life. In addition, each examined case and study did not always provide adequate information about the participants to determine the progression status of pwALS in their studies. Further research should be done with standardized measurements of quality of life to better compare different treatment modalities available. In addition, the small number of patients available in each study leads to increased variability in results. Due to the psychological nature of quality of life, cultural differences and variation in study populations can lead to differences in how quality of life is perceived. Further research should be done with large and more diverse populations to increase the strength of findings.

## Conclusion

ALS is an incurable progressive disease eventually leading to death, therefore treatment of pwALS should focus on increasing patient QOL. QOL in pwALS has been shown to be independent of physical function and more so dependent on psychological factors, except for dysarthria which leads to breakdown of communication inhibiting the maintenance of relationships, personal identity and autonomy which are determinants of psychological well-being and QOL. Therefore, the treatment of dysarthria and rehabilitation of communication should be a key goal in management of ALS. However, the current guidelines for management of communication in pwALS mainly encourage early referral to SLP and multidisciplinary clinics without recommendations on rehabilitation modalities. Further due to ALS’s progressive nature, the most common progression measurements do not effectively indicate the communication needs of a pwALS. Using the King’s and Milano torino staging systems, significant communication dysfunction milestones are captured, and can be used to organize different communication rehabilitation strategies to ensure consistent communication support throughout the changing needs of a pwALS through disease progression. Further a comparison based on quality of life and limitation of different rehabilitative modalities, will help to display options available and their effects and facilitate the optimization of QOL through management of communication. Before the onset of severe dysarthria or intelligibility, early-stage proactive measures such as education and voice banking can be used to improve QOL while utilizing natural speech which is the preferred communication method of most pwALS. Middle stage measures are AAC methods which can be used before pwALS enter the locked-in state and vary in accessibility and usability based on patient preferences and disease progression. Late stage AAC devices can be used within the locked-in state and provide functions unique to these devices. Devices such as ETCS are easy to use and more effective in increasing perceived QOL compared to middle stage interventions and should be introduced early in disease progression. Finally, treatment of dysarthria and communication rehabilitation should prioritize patient preferences and should be personalized to each patient, but a stage-based approach is an effective way of ensuring patients are offered the most optimal interventions for them throughout the disease process.

## Key Points

- ALS is a progressive and incurable disease, so treatment should focus on maximizing Quality of Life.
- Quality of Life in patients with ALS is independent of physical function except for dysarthria.
- Interventions for dysarthria such as Augmentative and alternative communication methods increase patient quality of life
- ALS progression varies between patients and interventions for speech rehabilitation should be chosen based on patients’ needs and preferences
- The most common ALS progression measure ALSFSRS-R does not adequately capture the communication needs of patients with ALS
- A combination of Kings, Milano Torino Staging systems capture disease milestones such as intelligibility and a locked in state which determine communication rehabilitation modality effectiveness
- Early interventions for speech rehabilitation are proactive and optimal before onset of intelligibility and are concurrently used with a patient’s natural speech.
- Middle stage interventions for speech rehabilitation vary in accessibility and usability based on patient preferences and disease progression and can only be used before onset of locked-in state.
- Late-stage interventions can be used after the onset of a locked-in state and are more effective in increasing perceived quality of life than middle stage interventions.

## References

[1] Vasta R, De Mattei F, Tafaro S, Changes to average survival of patients with amyotrophic lateral sclerosis (1995–2018): results from the Piemonte and Valle d’Aosta registry. Neurology 104 (2025): e213467. 40127392 10.1212/WNL.0000000000213467 PMC12012833

[2] Ball LJ, Beukelman DR, Pattee GL. Communication effectiveness of individuals with amyotrophic lateral sclerosis. J Commun Disord 37 (2004): 197–215. 15063143 10.1016/j.jcomdis.2003.09.002

[3] Goutman SA, Hardiman O, Al-Chalabi A, Recent advances in the diagnosis and prognosis of amyotrophic lateral sclerosis. Lancet Neurol 21 (2022): 480–493. 35334233 10.1016/S1474-4422(21)00465-8 PMC9513753

[4] Tzeplaeff L, Wilfling S, Requardt MV, Herdick M. Current state and future directions in the therapy of ALS. Cells 12 (2023): 1523. 37296644 10.3390/cells12111523 PMC10252394

[5] Card NS, Wairagkar M, Iacobacci C, An accurate and rapidly calibrating speech neuroprosthesis. N Engl J Med 391 (2024): 609–618. 39141853 10.1056/NEJMoa2314132 PMC11328962

[6] Hardiman O, Al-Chalabi A, Chio A, Amyotrophic lateral sclerosis. Nat Rev Dis Primers 3 (2017): 17085. 29052611 10.1038/nrdp.2017.85

[7] Van Damme P, Al-Chalabi A, Andersen PM, European Academy of Neurology (EAN) guideline on the management of amyotrophic lateral sclerosis in collaboration with European Reference Network for Neuromuscular Diseases (ERN EURO-NMD). Eur J Neurol 31 (2024): e16264. 38470068 10.1111/ene.16264 PMC11235832

[8] Simmons Z, Bremer BA, Robbins RA, Quality of life in ALS depends on factors other than strength and physical function. Neurology 55 (2000): 388–392. 10932273 10.1212/wnl.55.3.388

[9] Roach AR, Averill AJ, Segerstrom SC, The dynamics of quality of life in ALS patients and caregivers. Ann Behav Med 37 (2009): 197–206. 19350337 10.1007/s12160-009-9092-9

[10] Felgoise SH, Zaccheo V, Duff J, Verbal communication impacts quality of life in patients with amyotrophic lateral sclerosis. Amyotroph Lateral Scler Frontotemporal Degener 17 (2016): 179–183. 27094742 10.3109/21678421.2015.1125499

[11] Atkinson-Clement C, Letanneux A, Baille G, Psychosocial Impact of Dysarthria: The Patient-Reported Outcome as Part of the Clinical Management. Neurodegener Dis 19 (2019): 12–21. 31112944 10.1159/000499627

[12] McKelvey M, Evans DL, Kawai N, Communication styles of persons with ALS as recounted by surviving partners. Augment Altern Commun 28 (2012): 232–242. 23256855 10.3109/07434618.2012.737023

[13] Miller RG, Jackson CE, Kasarskis EJ, England JD, ; Quality Standards Subcommittee of the American Academy of Neurology. Practice parameter update: the care of the patient with amyotrophic lateral sclerosis: multidisciplinary care, symptom management, and cognitive/behavioral impairment (an evidence-based review): report of the Quality Standards Subcommittee of the American Academy of Neurology. Neurology 73 (2009): 1227–1233. 19822873 10.1212/WNL.0b013e3181bc01a4 PMC2764728

[14] Ball LJ, Beukelman DR, Pattee GL. Acceptance of Augmentative and Alternative Communication Technology by Persons with Amyotrophic Lateral Sclerosis. Augment Altern Commun 20 (2004): 113–122.

[15] Kimura F, Fujimura C, Ishida S, Progression rate of ALSFRS-R at time of diagnosis predicts survival time in ALS. Neurology 66 (2006): 265–267. 16434671 10.1212/01.wnl.0000194316.91908.8a

[16] Anab TH, Arishi AS, Hakami MA, The ability of King’s clinical staging and Milano-Torino (MiToS) functional staging in the prediction of amyotrophic lateral sclerosis (ALS) progression: A meta-analysis study. J Popul Ther Clin Pharmacol 31 (2004): 1315–1336.

[17] Plowman EK, Tabor LC, Wymer J, Pattee G. The evaluation of bulbar dysfunction in amyotrophic lateral sclerosis: survey of clinical practice patterns in the United States. Amyotroph Lateral Scler Frontotemporal Degener 18 (2017): 351–357. 28425762 10.1080/21678421.2017.1313868 PMC7001984

[18] Roche JC, Rojas-Garcia R, Scott KM, A proposed staging system for amyotrophic lateral sclerosis. Brain 135 (2012): 847–852. 22271664 10.1093/brain/awr351 PMC3286327

[19] Murphy J “I Prefer Contact This Close”: perceptions of AAC by people with motor neurone disease and their communication partners. Augment Altern Commun 20 (2004): 259–271.

[20] Hyppa-Martin J, Lilley J, Chen M, A large-scale comparison of two voice synthesis techniques on intelligibility, naturalness, preferences, and attitudes toward voices banked by individuals with amyotrophic lateral sclerosis. Augment Altern Commun 40 (2024): 31–45. 37791834 10.1080/07434618.2023.2262032

[21] Sixt Börjesson M, Hartelius L, Laakso K. Communicative Participation in People with Amyotrophic Lateral Sclerosis. Folia Phoniatr Logop 73 (2021): 101–108. 31918429 10.1159/000505022 PMC7949226

[22] Körner S, Sieniawski M, Kollewe K, Speech therapy and communication device: impact on quality of life and mood in patients with amyotrophic lateral sclerosis. Amyotroph Lateral Scler Frontotemporal Degener. 2013 Jan;14(1): 20–25. doi: 10.3109/17482968.2012.692382. Epub 2012 Aug 7. Erratum in: Amyotroph Lateral Scler Frontotemporal Degener. 2013 Apr;14(3):240. Siniawski, Michael [corrected to Sieniawski, Michael]. 22871079

[23] Londral A, Pinto A, Pinto S, Quality of life in amyotrophic lateral sclerosis patients and caregivers: Impact of assistive communication from early stages. Muscle Nerve 52 (2015): 933–941. 25808635 10.1002/mus.24659

[24] Maresca G, Pranio F, Naro A, Augmentative and alternative communication improves quality of life in the early stages of amyotrophic lateral sclerosis. Funct Neurol 34 (2019): 35–43. 31172938

[25] Connolly A, Bailey S, Lamont R, Factors associated with assistive technology prescription and acceptance in motor neurone disease. Disabil Rehabil Assist Technol 19 (2024): 2229–2238. 37897436 10.1080/17483107.2023.2272858

[26] Peters B, O’Brien K, Fried-Oken M. A recent survey of augmentative and alternative communication use and service delivery experiences of people with amyotrophic lateral sclerosis in the United States. Disabil Rehabil Assist Technol 19 (2024): 1121–1134. 36448513 10.1080/17483107.2022.2149866

[27] Murphy J Communication strategies of people with ALS and their partners. Amyotroph Lateral Scler Other Motor Neuron Disord 5 (2004): 121–126. 10.1080/14660820410020411 15204014

[28] Olmstead AJ, Lee J, Skrzat S, Everyday Communication Experiences of Persons With Amyotrophic Lateral Sclerosis and Their Caregivers: Implications for Novel Speech Interventions. Muscle Nerve. 2025 Jul; 72 (2025): 158–165. 40275673 10.1002/mus.28412 PMC12138489

[29] Munan M, Luth W, Genuis SK, Transitions in Amyotrophic Lateral Sclerosis: Patient and Caregiver Experiences. Can J Neurol Sci 48 (2021): 496–503. 33100231 10.1017/cjn.2020.240

[30] Cave R, Bloch S. Voice banking for people living with motor neurone disease: Views and expectations. Int J Lang Commun Disord. 2021 Jan; 56 (2021): 116–129. 33350040 10.1111/1460-6984.12588

[31] Cave R How People Living With Amyotrophic Lateral Sclerosis Use Personalized Automatic Speech Recognition Technology to Support Communication. J Speech Lang Hear Res 67 (2024): 4186–4202. 38991167 10.1044/2024_JSLHR-24-00097 PMC12379579

[32] Regondi S, Donvito G, Frontoni E, Artificial intelligence empowered voice generation for amyotrophic lateral sclerosis patients. Sci Rep 15 (2025): 1361. 39779800 10.1038/s41598-024-84728-y PMC11711320

[33] Costello J, Smith M. The BCH message banking process™, voice banking, and double-dipping™. Augment Altern Commun 37 (2021): 241–250. 35000518 10.1080/07434618.2021.2021554

[34] Donvito G, Greco LC, Lizio A, A pilot study of voice banking in amyotrophic lateral sclerosis patients. J Neurol Sci 459 (2024): 123018.

[35] Gonçalves F, Magalhães B. Effects of prolonged interruption of rehabilitation routines in amyotrophic lateral sclerosis patients. Palliat Support Care 20 (2022): 369–374. 33942709 10.1017/S1478951521000584

[36] Murphy J “I Prefer Contact This Close”: perceptions of AAC by people with motor neurone disease and their communication partners. Augment Altern Commun 20 (2004): 259–271.

[37] Beukelman D, Fager S, Nordness A. Communication Support for People with ALS. Neurol Res Int (2011): 714693. 21603029 10.1155/2011/714693 PMC3096454

[38] Soofi AY, Bello-Haas VD, Kho ME, The impact of rehabilitative interventions on quality of life: a qualitative evidence synthesis of personal experiences of individuals with amyotrophic lateral sclerosis. Qual Life Res 27 (2018): 845–856. 29204783 10.1007/s11136-017-1754-7

[39] Fried-Oken M, Fox L, Rau MT, Purposes of AAC device use for persons with ALS as reported by caregivers. Augment Altern Commun 22 (2006): 209–221. 17114164 10.1080/07434610600650276

[40] Spataro R, La Bella V. Determinants of quality of life in locked-in and complete locked-in ALS patients. J Neurol Sci 455 (2023): 121085.

[41] Yokoi D, Atsuta N, Watanabe H, ; JaCALS. Age of onset differentially influences the progression of regional dysfunction in sporadic amyotrophic lateral sclerosis. J Neurol 263 (2016): 1129–1136. 27083563 10.1007/s00415-016-8109-0

[42] Caligari M, Godi M, Giardini M, Development of a new high sensitivity mechanical switch for augmentative and alternative communication access in people with amyotrophic lateral sclerosis. J NeuroEngineering Rehabil 16 (2019): 152. 10.1186/s12984-019-0626-5 PMC6884866 31783763

[43] Caron J, Light J. “My World Has Expanded Even Though I’m Stuck at Home”: Experiences of Individuals With Amyotrophic Lateral Sclerosis Who Use Augmentative and Alternative Communication and Social Media. Am J Speech Lang Pathol 24 (2015): 680–695. 26254447 10.1044/2015_AJSLP-15-0010

[44] Lancioni GE, Singh NN, O’Reilly MF, A man with amyotrophic lateral sclerosis uses a mouth pressure microswitch to operate a text messaging system with a word prediction function. Dev Neurorehabil. 2013 Oct;16(5): 315–20. 24020877 10.3109/17518423.2012.731086

[45] Lancioni GE, Ferlisi G, Zullo V, Two men with advanced amyotrophic lateral sclerosis operate a computer-aided television system through mouth or throat microswitches. Percept Mot Skills 118 (2014): 883–889. 25068751 10.2466/15.PMS.118k24w2

[46] Lancioni GE, Singh NN, O’Reilly MF, A voice-sensitive microswitch for a man with amyotrophic lateral sclerosis and pervasive motor impairment. Disabil Rehabil Assist Technol 9 (2013): 260–263. 23597318 10.3109/17483107.2013.785037

[47] Lancioni GE, Singh NN, O’Reilly MF, A basic technology-aided programme for leisure and communication of persons with advanced amyotrophic lateral sclerosis: performance and social rating. Disabil Rehabil Assist Technol 12 (2015) :145–152. 26699449 10.3109/17483107.2015.1104561

[48] Harris D, Goren M. The ERICA eye gaze system versus manual letter board to aid communication in ALS/MND. Br J Neurosci Nurs 5 (2009): 227–230.

[49] Caligari M, Godi M, Guglielmetti S, Eye tracking communication devices in amyotrophic lateral sclerosis: impact on disability and quality of life. Amyotroph Lateral Scler Frontotemporal Degener 14 (2013): 546–552. 23834069 10.3109/21678421.2013.803576

[50] Cai S, Venugopalan S, Seaver K, Using large language models to accelerate communication for eye gaze typing users with ALS. Nat Commun 15 (2024): 9449. 39487163 10.1038/s41467-024-53873-3 PMC11530652

[51] Linse K, Rüger W, Joos M, Usability of eyetracking computer systems and impact on psychological wellbeing in patients with advanced amyotrophic lateral sclerosis. Amyotroph Lateral Scler Frontotemporal Degener 19 (2018): 212–219. 29092645 10.1080/21678421.2017.1392576

[52] Linse K, Rüger W, Joos M, Eye-tracking-based assessment suggests preserved well-being in locked-in patients. Ann Neurol 81 (2017): 310–315. 28074605 10.1002/ana.24871

[53] Hwang CS, Weng HH, Wang LF, An eye-tracking assistive device improves the quality of life for ALS patients and reduces the caregivers’ burden. J Mot Behav 46 (2014): 233–238. 24731126 10.1080/00222895.2014.891970

[54] Spataro R, Ciriacono M, Manno C, La Bella V. The eye-tracking computer device for communication in amyotrophic lateral sclerosis. Acta Neurol Scand. 2014 Jul; 130 (2014): 40–45. 24350578 10.1111/ane.12214

[55] Peters B, Bedrick S, Dudy S, SSVEP BCI and Eye Tracking Use by Individuals With Late-Stage ALS and Visual Impairments. Front Hum Neurosci 14 (2020): 595890. 33328941 10.3389/fnhum.2020.595890 PMC7715037

[56] Käthner I, Kübler A, Halder S. Comparison of eye tracking, electrooculography and an auditory brain-computer interface for binary communication: a case study with a participant in the locked-in state. J Neuroeng Rehabil 12 (2015): 76. 26338101 10.1186/s12984-015-0071-z PMC4560087

[57] Vansteensel MJ, Leinders S, Branco MP, Longevity of a Brain-Computer Interface for Amyotrophic Lateral Sclerosis. N Engl J Med 391 (2024): 619–626. 39141854 10.1056/NEJMoa2314598 PMC11395392

[58] Wairagkar M, Card NS, Singer-Clark T, An instantaneous voice synthesis neuroprosthesis. bioRxiv [Preprint]. 2024 Sep 20:2024.08.14.607690. Update in: Nature 644 (2025): 145–152. 10.1038/s41586-025-09127-3 PMC12369848 40506548

[59] Münßinger JI, Halder S, Kleih SC, Brain Painting: First Evaluation of a New Brain-Computer Interface Application with ALS-Patients and Healthy Volunteers. Front Neurosci 4 (2010): 182. 21151375 10.3389/fnins.2010.00182 PMC2996245

[60] Holz EM, Botrel L, Kaufmann T, Long-term independent brain-computer interface home use improves quality of life of a patient in the locked-in state: a case study. Arch Phys Med Rehabil 96 (2015): S16–26. 25721543 10.1016/j.apmr.2014.03.035

[61] Huggins JE, Wren PA, Gruis KL. What would brain-computer interface users want? Opinions and priorities of potential users with amyotrophic lateral sclerosis. Amyotroph Lateral Scler 12 (2011): 318–324. 21534845 10.3109/17482968.2011.572978 PMC3286341

[62] Kageyama Y, He X, Shimokawa T, Nationwide survey of 780 Japanese patients with amyotrophic lateral sclerosis: their status and expectations from brain-machine interfaces. J Neurol 267 (2020): 2932–2940. 32488296 10.1007/s00415-020-09903-3

[63] Sellers EW, Vaughan TM, Wolpaw JR. A brain-computer interface for long-term independent home use. Amyotroph Lateral Scler 11 (2010): 449–455. 20583947 10.3109/17482961003777470

[64] Bublitz SK, Eham M, Ellrott H, Homecare amyotrophic lateral sclerosis (ALS): A multidisciplinary, home-based model of care for patients with ALS and their caregivers. Muscle Nerve 69 (2024): e28218. 10.1002/mus.28218 39073146

